# Supplementary material for: The relationship between cancer associated fibroblasts biomarkers and prognosis of breast cancer: a systematic review and meta-analysis
Source: PeerJ. 2024 Feb 23;12:e16958. doi: 10.7717/peerj.16958 (PMC10896086; doi:10.7717/peerj.16958)
Supplement: Supplemental Information 1 [file peerj-12-16958-s001.pdf]

National Institute for Health Research

UNIVERSITY of York
Centre for Reviews and Dissemination

Systematic review

A list of fields that can be edited in an update can be found here

1. * Review title. [1 change]

Give the title of the review in English

The Relationship between Cancer associated Fibroblasts Markers and Prognosis of Breast Cancer: A Systematic Review and Meta-Analysis

2. Original language title.

For reviews in languages other than English, give the title in the original language. This will be displayed with the English language title.

3. * Anticipated or actual start date.

Give the date the systematic review started or is expected to start.

09/07/2023

4. * Anticipated completion date. [1 change]

Give the date by which the review is expected to be completed.

27/09/2023

5. * Stage of review at time of this submission. [1 change]

This field uses answers to initial screening questions. It cannot be edited until after registration.

Tick the boxes to show which review tasks have been started and which have been completed.

Update this field each time any amendments are made to a published record.

The review has not yet started: No

Review stage	Started	Completed
Preliminary searches	Yes	Yes
Piloting of the study selection process	Yes	Yes
Formal screening of search results against eligibility criteria	Yes	Yes
Data extraction	Yes	Yes
Risk of bias (quality) assessment	Yes	Yes
Data analysis	Yes	Yes

Provide any other relevant information about the stage of the review here.

6. * Named contact.

The named contact is the guarantor for the accuracy of the information in the register record. This may be any member of the review team.

Cui Meimei

Email salutation (e.g. "Dr Smith" or "Joanne") for correspondence:

Ms Meimei

7. * Named contact email.

Give the electronic email address of the named contact.

cuimm9989@163.com

8. Named contact address

Give the full institutional/organisational postal address for the named contact.

weifang medical university, guangwen street, kuiwen district, weifang city, shandong province

9. Named contact phone number.

Give the telephone number for the named contact, including international dialling code.

19990809

10. * Organisational affiliation of the review.

Full title of the organisational affiliations for this review and website address if available. This field may be

completed as 'None' if the review is not affiliated to any organisation.

Department of Pathology, Weifang Medical University, Weifang, Shandong China

Organisation web address:

11. * Review team members and their organisational affiliations. [1 change]

Give the personal details and the organisational affiliations of each member of the review team. Affiliation refers to groups or organisations to which review team members belong. **NOTE: email and country now MUST be entered for each person, unless you are amending a published record.**

Ms cui meimei. Department of Pathology, Weifang Medical University, Weifang, Shandong China
Dong Hao. Department of Pathology, School of Basic Medical Sciences, Weifang Medical University, Weifang, 261041, China
Duan Wanli. Department of Pathology, School of Basic Medical Sciences, Weifang Medical University, Weifang, 261041, China
Wang Xuejie. Department of Pathology, School of Basic Medical Sciences, Weifang Medical University, Weifang, 261041, China
Liu Yongping. Department of Pathology, School of Basic Medical Sciences, Weifang Medical University, Weifang, 261041, China
Shi Lihong. School of Rehabilitation Medicine, Weifang Medical University, Weifang, 261041, China
Zhang Baogang. Department of Pathology, School of Basic Medical Sciences, Weifang Medical University, Weifang, 261041, China

12. * Funding sources/sponsors.

Details of the individuals, organizations, groups, companies or other legal entities who have funded or sponsored the review.

This work was supported by the National Natural Scientific Foundation of China

Grant number(s)

State the funder, grant or award number and the date of award

81872163

13. * Conflicts of interest.

List actual or perceived conflicts of interest (financial or academic).

None

14. Collaborators. [1 change]

Give the name and affiliation of any individuals or organisations who are working on the review but who are not listed as review team members. **NOTE: email and country must be completed for each person, unless you are amending a published record.**

Luo Limei. Shandong Provincial Maternal and Child Health Care Hospital Affiliated to Qingdao University,

Jinan, 250014, China.

15. * Review question.

State the review question(s) clearly and precisely. It may be appropriate to break very broad questions down into a series of related more specific questions. Questions may be framed or refined using PI(E)COS or similar where relevant.

Cancer-associated fibroblasts (CAFs) are key components of the tumor microenvironment (TME); however, the role of CAFs heterogeneity in breast cancer (BC) prognosis remains unclear. This study provides a deeper understanding of CAFs heterogeneity and the biological characteristics of BC.

16. * Searches. [1 change]

State the sources that will be searched (e.g. Medline). Give the search dates, and any restrictions (e.g. language or publication date). Do NOT enter the full search strategy (it may be provided as a link or attachment below.)

We searched the PubMed, Web of Science, Embase and Cochrane Library from their inception dates to September, 2023.

17. URL to search strategy.

Upload a file with your search strategy, or an example of a search strategy for a specific database, (including the keywords) in pdf or word format. In doing so you are consenting to the file being made publicly accessible. Or provide a URL or link to the strategy. Do NOT provide links to your search **results**.

Alternatively, upload your search strategy to CRD in pdf format. Please note that by doing so you are consenting to the file being made publicly accessible.

Do not make this file publicly available until the review is complete

18. * Condition or domain being studied.

Give a short description of the disease, condition or healthcare domain being studied in your systematic review.

The latest global cancer data for 2020 shows that there were as many as 2.26 million new cases of breast cancer (BC) worldwide, surpassing lung cancer's 2.2 million cases, making BC the leading cancer worldwide. Despite recent advances in treatment, the results remain poor, and there is an urgent need for innovative methods for BC treatment to reduce the recurrence and mortality of BC.

19. * Participants/population.

Specify the participants or populations being studied in the review. The preferred format includes details of both inclusion and exclusion criteria.

Breast cancer patients

20. * Intervention(s), exposure(s).

Give full and clear descriptions or definitions of the interventions or the exposures to be reviewed. The preferred format includes details of both inclusion and exclusion criteria.

Not Applicable

21. * Comparator(s)/control.

Where relevant, give details of the alternatives against which the intervention/exposure will be compared (e.g. another intervention or a non-exposed control group). The preferred format includes details of both inclusion and exclusion criteria.

Not Applicable

22. * Types of study to be included.

Give details of the study designs (e.g. RCT) that are eligible for inclusion in the review. The preferred format includes both inclusion and exclusion criteria. If there are no restrictions on the types of study, this should be stated.

cohort study

23. Context.

Give summary details of the setting or other relevant characteristics, which help define the inclusion or exclusion criteria.

24. * Main outcome(s).

Give the pre-specified main (most important) outcomes of the review, including details of how the outcome is defined and measured and when these measurement are made, if these are part of the review inclusion criteria.

Describe the relationship between CAFs markers and patient prognosis, mainly conducting meta-analysis on two or more studies, resulting in HR and 95% CI

Measures of effect

Please specify the effect measure(s) for you main outcome(s) e.g. relative risks, odds ratios, risk difference, and/or 'number needed to treat.

25. * Additional outcome(s).

List the pre-specified additional outcomes of the review, with a similar level of detail to that required for main outcomes. Where there are no additional outcomes please state ‘None’ or ‘Not applicable’ as appropriate to the review

Results analysis will base on the function of the biomarkers

Measures of effect

Please specify the effect measure(s) for you additional outcome(s) e.g. relative risks, odds ratios, risk

difference, and/or 'number needed to treat.

26. * Data extraction (selection and coding).

Describe how studies will be selected for inclusion. State what data will be extracted or obtained. State how this will be done and recorded.

Three researchers independently will conduct literature screening, data extraction, and quality assessment and cross-checked="checked" value="1" each other's work. Disputes will be resolved through consultation, consensus, or discussion with a third author. A literature flowchart and data extraction form will be created, and the literature extraction content will include the following: first author, publication year, number of patients, median age, follow-up time, methods will be use to quantify fibroblasts, cutoff values will use to determine the density of these cells, and data on OS, DFS, DSS, DFS, PFS, and MFS. If an article only provides a KM curve without relevant HRs, we will use Engauge Digitizer 4.1 (Mark Mitchell) to extract the missing HRs from the survival curve data and analyze the data using the tools developed by Tierney et al.

27. * Risk of bias (quality) assessment.

State which characteristics of the studies will be assessed and/or any formal risk of bias/quality assessment tools that will be used.

We will use funnel plots and Begg's test to assess publication bias.

28. * Strategy for data synthesis.

Describe the methods you plan to use to synthesise data. This **must not be generic text** but should be **specific to your review** and describe how the proposed approach will be applied to your data. If meta-analysis is planned, describe the models to be used, methods to explore statistical heterogeneity, and software package to be used.

After extracting all relevant data, we first group similar survival outcomes into three categories: OS/DSS, RFS/DFS/MFS, and PFS. However, as suggested by Riley et al, we consider both univariate and multivariate analysis-derived HRs separately. Using the inverse variance method, we creat weighted HRs with 95% CIs and P for random-effects models, based on at least two different cohorts of individual markers with the same outcome group and analysis method. Forest plots for individual markers wil be generated using R 4.2.2 (College Station, Texas). An HRs of 1 indicat no association between the exposure factor and the outcome event, HRs 1 indicat a shorter survival time for the exposed group (risk factor), and HRs 1 indicat a longer survival time for the exposed group (protective factor). Heterogeneity will be assessed by calculating I^2 and ?2 values, and P will be generated to evaluate the statistical significance of heterogeneity. For I^2 index, where I^2 50% indicate low heterogeneity among studies and a fixed-effects model will be applied, while I^2 50% indicated high heterogeneity and a random-effects model will be adopted.

29. * Analysis of subgroups or subsets.

State any planned investigation of ‘subgroups’. Be clear and specific about which type of study or participant will be included in each group or covariate investigated. State the planned analytic approach.

Due to the substantial heterogeneity in some of the results, we will conduct a subgroup analysis to explore the sources of heterogeneity.

30. * Type and method of review.

Select the type of review, review method and health area from the lists below.

Type of review

Cost effectiveness
No

Diagnostic
No

Epidemiologic
No

Individual patient data (IPD) meta-analysis
No

Intervention
No

Living systematic review
No

Meta-analysis
Yes

Methodology
No

Narrative synthesis
No

Network meta-analysis
No

Pre-clinical
No

Prevention
No

Prognostic
Yes

Prospective meta-analysis (PMA)
No

Review of reviews
No

Service delivery

No

Synthesis of qualitative studies

No

Systematic review

Yes

Other

No

Health area of the review

Alcohol/substance misuse/abuse

No

Blood and immune system

No

Cancer

Yes

Cardiovascular

No

Care of the elderly

No

Child health

No

Complementary therapies

No

COVID-19

No

Crime and justice

No

Dental

No

Digestive system

No

Ear, nose and throat

No

Education

No

Endocrine and metabolic disorders

No

Eye disorders

No

General interest

No

Genetics

No

Health inequalities/health equity

No

Infections and infestations

No

International development

No

Mental health and behavioural conditions

No

Musculoskeletal

No

Neurological

No

Nursing

No

Obstetrics and gynaecology

No

Oral health

No

Palliative care

No

Perioperative care

No

Physiotherapy

No

Pregnancy and childbirth

No

Public health (including social determinants of health)

No

Rehabilitation

No

Respiratory disorders

No

Service delivery

No

Skin disorders

No

Social care

No

Surgery

No

Tropical Medicine

No

Urological

No

Wounds, injuries and accidents

No

Violence and abuse

No

31. Language.

Select each language individually to add it to the list below, use the bin icon to remove any added in error.

English

There is not an English language summary

32. * Country.

Select the country in which the review is being carried out. For multi-national collaborations select all the countries involved.

China

33. Other registration details.

Name any other organisation where the systematic review title or protocol is registered (e.g. Campbell, or The Joanna Briggs Institute) together with any unique identification number assigned by them. If extracted data will be stored and made available through a repository such as the Systematic Review Data Repository (SRDR), details and a link should be included here. If none, leave blank.

34. Reference and/or URL for published protocol.

If the protocol for this review is published provide details (authors, title and journal details, preferably in

Vancouver format)

Add web link to the published protocol.

Or, upload your published protocol here in pdf format. Note that the upload will be publicly accessible.

No I do not make this file publicly available until the review is complete

Please note that the information required in the PROSPERO registration form must be completed in full even if access to a protocol is given.

35. Dissemination plans.

Do you intend to publish the review on completion?

No

Give brief details of plans for communicating review findings.?

36. Keywords.

Give words or phrases that best describe the review. Separate keywords with a semicolon or new line. Keywords help PROSPERO users find your review (keywords do not appear in the public record but are included in searches). Be as specific and precise as possible. Avoid acronyms and abbreviations unless these are in wide use.

Breast cancer; Cancer associated fibroblasts; Meta-analysis; Prognostic markers; Tumor microenvironment

37. Details of any existing review of the same topic by the same authors.

If you are registering an update of an existing review give details of the earlier versions and include a full bibliographic reference, if available.

38. * Current review status. [1 change]

Update review status when the review is completed and when it is published.New registrations must be ongoing so this field is not editable for initial submission.

Please provide anticipated publication date

Review_Completed_not_published

39. Any additional information.

Provide any other information relevant to the registration of this review.

40. Details of final report/publication(s) or preprints if available.

Leave empty until publication details are available OR you have a link to a preprint (NOTE: this field is not editable for initial submission). List authors, title and journal details preferably in Vancouver format.

Give the link to the published review or preprint.
