# Supplementary material for: The relationship between cancer associated fibroblasts biomarkers and prognosis of breast cancer: a systematic review and meta-analysis
Source: PeerJ. 2024 Feb 23;12:e16958. doi: 10.7717/peerj.16958 (PMC10896086; doi:10.7717/peerj.16958)
Supplement: Supplemental Information 2 [file peerj-12-16958-s002.docx]

| Search strategy | | | |
| --- | --- | --- | --- |
| PubMed | 1101 |  |  |
| Search term | MESH | free words | search strategy |
| Cancer-Associated Fibroblasts | Cancer-Associated Fibroblasts | Cancer-Associated Fibroblast  Fibroblast, Cancer-Associated  Fibroblasts, Cancer-Associated  Cancer Associated Fibroblasts  Cancer Associated Fibroblast  Fibroblast, Cancer Associated  Fibroblasts, Cancer Associated  Tumor-Associated Fibroblasts  Fibroblast, Tumor-Associated  Fibroblasts, Tumor-Associated  Tumor Associated Fibroblasts  Tumor-Associated Fibroblast | Search: **("Cancer-Associated Fibroblasts"[Mesh]) OR ((((((((((((Cancer-Associated Fibroblast[Title/Abstract]) OR (Fibroblast, Cancer-Associated[Title/Abstract])) OR (Fibroblasts, Cancer-Associated[Title/Abstract])) OR (Cancer Associated Fibroblasts[Title/Abstract])) OR (Cancer Associated Fibroblast[Title/Abstract])) OR (Fibroblast, Cancer Associated[Title/Abstract])) OR (Fibroblasts, Cancer Associated[Title/Abstract])) OR (Tumor-Associated Fibroblasts[Title/Abstract])) OR (Fibroblast, Tumor-Associated[Title/Abstract])) OR (Fibroblasts, Tumor-Associated[Title/Abstract])) OR (Tumor Associated Fibroblasts[Title/Abstract])) OR (Tumor-Associated Fibroblast[Title/Abstract]))** |
| Breast cancer |  | Breast Neoplasm  Neoplasm, Breast  Neoplasms, Breast  Breast Tumors  Breast Tumor  Tumor, Breast  Tumors, Breast  Breast Cancer  Cancer, Breast  Malignant Tumor of Breast  Breast Malignant Tumor  Breast Malignant Tumors  Cancer of the Breast  Cancer of Breast  Malignant Neoplasm of Breast  Breast Malignant Neoplasm  Breast Malignant Neoplasms  Mammary Cancer  Cancer, Mammary  Cancers, Mammary  Mammary Cancers  Mammary Carcinoma, Human  Carcinoma, Human Mammary  Carcinomas, Human Mammary  Human Mammary Carcinomas  Mammary Carcinomas, Human  Human Mammary Carcinoma  Mammary Neoplasms, Human  Human Mammary Neoplasm  Human Mammary Neoplasms  Neoplasm, Human Mammary  Neoplasms, Human Mammary  Mammary Neoplasm, Human  Breast Carcinoma  Breast Carcinomas  Carcinoma, Breast  Carcinomas, Breast | Search: **("Breast Neoplasms"[Mesh]) OR (((((((((((((((((((((((((((((((((((((Breast Neoplasm[Title/Abstract]) OR (Neoplasm, Breast[Title/Abstract])) OR (Neoplasms, Breast[Title/Abstract])) OR (Breast Tumors[Title/Abstract])) OR (Breast Tumor[Title/Abstract])) OR (Tumor, Breast[Title/Abstract])) OR (Tumors, Breast[Title/Abstract])) OR (Breast Cancer[Title/Abstract])) OR (Cancer, Breast[Title/Abstract])) OR (Malignant Tumor of Breast[Title/Abstract])) OR (Breast Malignant Tumor[Title/Abstract])) OR (Breast Malignant Tumors[Title/Abstract])) OR (Cancer of the Breast[Title/Abstract])) OR (Cancer of Breast[Title/Abstract])) OR (Malignant Neoplasm of Breast[Title/Abstract])) OR (Breast Malignant Neoplasm[Title/Abstract])) OR (Breast Malignant Neoplasms[Title/Abstract])) OR (Mammary Cancer[Title/Abstract])) OR (Cancer, Mammary[Title/Abstract])) OR (Cancers, Mammary[Title/Abstract])) OR (Mammary Cancers[Title/Abstract])) OR (Mammary Carcinoma, Human[Title/Abstract])) OR (Carcinoma, Human Mammary[Title/Abstract])) OR (Carcinomas, Human Mammary[Title/Abstract])) OR (Human Mammary Carcinomas[Title/Abstract])) OR (Mammary Carcinomas, Human[Title/Abstract])) OR (Human Mammary Carcinoma[Title/Abstract])) OR (Mammary Neoplasms, Human[Title/Abstract])) OR (Human Mammary Neoplasm[Title/Abstract])) OR (Human Mammary Neoplasms[Title/Abstract])) OR (Neoplasm, Human Mammary[Title/Abstract])) OR (Neoplasms, Human Mammary[Title/Abstract])) OR (Mammary Neoplasm, Human[Title/Abstract])) OR (Breast Carcinoma[Title/Abstract])) OR (Breast Carcinomas[Title/Abstract])) OR (Carcinoma, Breast[Title/Abstract])) OR (Carcinomas, Breast[Title/Abstract]))** |
| Search: **(("Cancer-Associated Fibroblasts"[Mesh]) OR ((((((((((((Cancer-Associated Fibroblast[Title/Abstract]) OR (Fibroblast, Cancer-Associated[Title/Abstract])) OR (Fibroblasts, Cancer-Associated[Title/Abstract])) OR (Cancer Associated Fibroblasts[Title/Abstract])) OR (Cancer Associated Fibroblast[Title/Abstract])) OR (Fibroblast, Cancer Associated[Title/Abstract])) OR (Fibroblasts, Cancer Associated[Title/Abstract])) OR (Tumor-Associated Fibroblasts[Title/Abstract])) OR (Fibroblast, Tumor-Associated[Title/Abstract])) OR (Fibroblasts, Tumor-Associated[Title/Abstract])) OR (Tumor Associated Fibroblasts[Title/Abstract])) OR (Tumor-Associated Fibroblast[Title/Abstract]))) AND (("Breast Neoplasms"[Mesh]) OR (((((((((((((((((((((((((((((((((((((Breast Neoplasm[Title/Abstract]) OR (Neoplasm, Breast[Title/Abstract])) OR (Neoplasms, Breast[Title/Abstract])) OR (Breast Tumors[Title/Abstract])) OR (Breast Tumor[Title/Abstract])) OR (Tumor, Breast[Title/Abstract])) OR (Tumors, Breast[Title/Abstract])) OR (Breast Cancer[Title/Abstract])) OR (Cancer, Breast[Title/Abstract])) OR (Malignant Tumor of Breast[Title/Abstract])) OR (Breast Malignant Tumor[Title/Abstract])) OR (Breast Malignant Tumors[Title/Abstract])) OR (Cancer of the Breast[Title/Abstract])) OR (Cancer of Breast[Title/Abstract])) OR (Malignant Neoplasm of Breast[Title/Abstract])) OR (Breast Malignant Neoplasm[Title/Abstract])) OR (Breast Malignant Neoplasms[Title/Abstract])) OR (Mammary Cancer[Title/Abstract])) OR (Cancer, Mammary[Title/Abstract])) OR (Cancers, Mammary[Title/Abstract])) OR (Mammary Cancers[Title/Abstract])) OR (Mammary Carcinoma, Human[Title/Abstract])) OR (Carcinoma, Human Mammary[Title/Abstract])) OR (Carcinomas, Human Mammary[Title/Abstract])) OR (Human Mammary Carcinomas[Title/Abstract])) OR (Mammary Carcinomas, Human[Title/Abstract])) OR (Human Mammary Carcinoma[Title/Abstract])) OR (Mammary Neoplasms, Human[Title/Abstract])) OR (Human Mammary Neoplasm[Title/Abstract])) OR (Human Mammary Neoplasms[Title/Abstract])) OR (Neoplasm, Human Mammary[Title/Abstract])) OR (Neoplasms, Human Mammary[Title/Abstract])) OR (Mammary Neoplasm, Human[Title/Abstract])) OR (Breast Carcinoma[Title/Abstract])) OR (Breast Carcinomas[Title/Abstract])) OR (Carcinoma, Breast[Title/Abstract])) OR (Carcinomas, Breast[Title/Abstract])))** | | | |

| WOS | 5024 |  |
| --- | --- | --- |
| Cancer-Associated Fibroblasts | Cancer-Associated Fibroblast  Fibroblast, Cancer-Associated  Fibroblasts, Cancer-Associated  Cancer Associated Fibroblasts  Cancer Associated Fibroblast  Fibroblast, Cancer Associated  Fibroblasts, Cancer Associated  Tumor-Associated Fibroblasts  Fibroblast, Tumor-Associated  Fibroblasts, Tumor-Associated  Tumor Associated Fibroblasts  Tumor-Associated Fibroblast | **(((((((((((TS=(Cancer-Associated Fibroblast)) OR TS=(Fibroblast, Cancer-Associated)) OR TS=(Fibroblasts, Cancer-Associated)) OR TS=(Cancer Associated Fibroblasts)) OR TS=(Cancer Associated Fibroblast)) OR TS=(Fibroblast, Cancer Associated)) OR TS=(Fibroblasts, Cancer Associated)) OR TS=(Tumor-Associated Fibroblasts)) OR TS=(Fibroblast, Tumor-Associated)) OR TS=(Fibroblasts, Tumor-Associated)) OR TS=(Tumor Associated Fibroblasts)) OR TS=(Tumor-Associated Fibroblast)** |
| Breast cancer | Breast Neoplasm  Neoplasm, Breast  Neoplasms, Breast  Breast Tumors  Breast Tumor  Tumor, Breast  Tumors, Breast  Breast Cancer  Cancer, Breast  Malignant Tumor of Breast  Breast Malignant Tumor  Breast Malignant Tumors  Cancer of the Breast  Cancer of Breast  Malignant Neoplasm of Breast  Breast Malignant Neoplasm  Breast Malignant Neoplasms  Mammary Cancer  Cancer, Mammary  Cancers, Mammary  Mammary Cancers  Mammary Carcinoma, Human  Carcinoma, Human Mammary  Carcinomas, Human Mammary  Human Mammary Carcinomas  Mammary Carcinomas, Human  Human Mammary Carcinoma  Mammary Neoplasms, Human  Human Mammary Neoplasm  Human Mammary Neoplasms  Neoplasm, Human Mammary  Neoplasms, Human Mammary  Mammary Neoplasm, Human  Breast Carcinoma  Breast Carcinomas  Carcinoma, Breast  Carcinomas, Breast | **((((((((((((((((((((((((((((((((((((TS=(Breast Neoplasm)) OR TS=(Neoplasm, Breast)) OR TS=(Neoplasms, Breast)) OR TS=(Breast Tumors)) OR TS=(Breast Tumor)) OR TS=(Tumor, Breast)) OR TS=(Tumors, Breast)) OR TS=(Breast Cancer)) OR TS=(Cancer, Breast)) OR TS=(Malignant Tumor of Breast)) OR TS=(Breast Malignant Tumor)) OR TS=(Breast Malignant Tumors)) OR TS=(Cancer of the Breast)) OR TS=(Cancer of Breast)) OR TS=(Malignant Neoplasm of Breast)) OR TS=(Breast Malignant Neoplasm)) OR TS=(Breast Malignant Neoplasms)) OR TS=(Mammary Cancer)) OR TS=(Cancer, Mammary)) OR TS=(Cancers, Mammary)) OR TS=(Mammary Cancers)) OR TS=(Mammary Carcinoma, Human)) OR TS=(Carcinoma, Human Mammary)) OR TS=(Carcinomas, Human Mammary)) OR TS=(Human Mammary Carcinomas)) OR TS=(Mammary Carcinomas, Human)) OR TS=(Human Mammary Carcinoma)) OR TS=(Mammary Neoplasms, Human)) OR TS=(Human Mammary Neoplasm)) OR TS=(Human Mammary Neoplasms)) OR TS=(Neoplasm, Human Mammary)) OR TS=(Neoplasms, Human Mammary)) OR TS=(Mammary Neoplasm, Human)) OR TS=(Breast Carcinoma)) OR TS=(Breast Carcinomas)) OR TS=(Carcinoma, Breast)) OR TS=(Carcinomas, Breast)** |

| Cochrane | 28 |  |  |
| --- | --- | --- | --- |
| Cancer Associated Fibroblasts | [Cancer-Associated Fibroblasts](http://www-cochranelibrary-com-443.bjmu.ilibs.cn/advanced-search/mesh#0) | Fibroblasts, Cancer-Associated;  Tumor-Associated Fibroblasts;  Cancer Associated Fibroblasts;  Cancer Associated Fibroblast;  Cancer-Associated Fibroblast;  Fibroblast, Cancer-Associated;  Tumor Associated Fibroblasts;  Fibroblast, Tumor-Associated;  Fibroblasts, Cancer Associated;  Fibroblast, Cancer Associated;  Tumor-Associated Fibroblast;  Fibroblasts, Tumor-Associated |  |
| Breast cancer | [Breast Neoplasms](http://www-cochranelibrary-com-443.ccmu.goodjob666.com/advanced-search/mesh#0) | Human Mammary Carcinoma;  Carcinoma, Human Mammary;  Carcinomas, Human Mammary;  Mammary Carcinomas, Human;  Human Mammary Carcinomas;  Mammary Carcinoma, Human;  Mammary Neoplasm, Human;  Neoplasms, Human Mammary;  Human Mammary Neoplasm;  Human Mammary Neoplasms;  Mammary Neoplasms, Human;  Neoplasm, Human Mammary;  Breast Malignant Tumor;  Breast Cancer;  Cancer of the Breast;  Malignant Tumor of Breast;  Breast Malignant Neoplasms;  Cancers, Mammary;  Cancer of Breast;  Malignant Neoplasm of Breast;  Cancer, Mammary;  Breast Malignant Tumors;  Breast Malignant Neoplasm;  Mammary Cancers;  Mammary Cancer;  Cancer, Breast;  Neoplasm, Breast;  Breast Tumors;  Tumor, Breast;  Breast Tumor;  Tumors, Breast;  Neoplasms, Breast;  Breast Neoplasm;  Breast Carcinomas;  Carcinoma, Breast;  Carcinomas, Breast;  Breast Carcinoma |  |

| Embase | 1903 |  |  |
| --- | --- | --- | --- |
| Cancer Associated Fibroblasts | cancer associated fibroblasts;  cancer-associated fibroblast;  cancer-associated fibroblasts;  tumor associated fibroblast;  tumor associated fibroblasts;  tumor-associated fibroblast;  tumor-associated fibroblasts |  | 'cancer associated fibroblast'/exp  'cancer associated fibroblasts':ti,ab,kw OR 'cancer-associated fibroblast':ti,ab,kw OR 'cancer-associated fibroblasts':ti,ab,kw OR 'tumor associated fibroblast':ti,ab,kw OR 'tumor associated fibroblasts':ti,ab,kw OR 'tumor-associated fibroblast':ti,ab,kw OR 'tumor-associated fibroblasts':ti,ab,kw |
| Breast cancer | breast gland cancer;  breast gland neoplasm;  breast malignancies;  breast malignancy;  breast tumor malignant;  Ca breast;  cancer in the mammary gland;  cancer of the breast;  cancer of the mammary gland;  cancer, breast;  malignancies of the breast;  malignancy of the breast;  malignant breast neoplasm;  malignant breast tumor;  malignant neoplasm of the breast;  malignant tumor of the breast;  mamma cancer;  mammary cancer;  mammary gland cancer;  mammary gland malignancy;  mammary malignancies;  mammary malignancy |  | 'breast cancer'/exp  'breast gland cancer':ti,ab,kw OR 'breast gland neoplasm':ti,ab,kw OR 'breast malignancies':ti,ab,kw OR 'breast malignancy':ti,ab,kw OR 'breast tumor malignant':ti,ab,kw OR 'ca breast':ti,ab,kw OR 'cancer in the mammary gland':ti,ab,kw OR 'cancer of the breast':ti,ab,kw OR 'cancer of the mammary gland':ti,ab,kw OR 'cancer, breast':ti,ab,kw OR 'malignancies of the breast':ti,ab,kw OR 'malignancy of the breast':ti,ab,kw OR 'malignant breast neoplasm':ti,ab,kw OR 'malignant breast tumor':ti,ab,kw OR 'malignant neoplasm of the breast':ti,ab,kw OR 'malignant tumor of the breast':ti,ab,kw OR 'mamma cancer':ti,ab,kw OR 'mammary cancer':ti,ab,kw OR 'mammary gland cancer':ti,ab,kw OR 'mammary gland malignancy':ti,ab,kw OR 'mammary malignancies':ti,ab,kw OR 'mammary malignancy':ti,ab,kw |

All：8056
