# Supplementary material for: The relationship between cancer associated fibroblasts biomarkers and prognosis of breast cancer: a systematic review and meta-analysis
Source: PeerJ. 2024 Feb 23;12:e16958. doi: 10.7717/peerj.16958 (PMC10896086; doi:10.7717/peerj.16958)
Supplement: Supplemental Information 6 [file peerj-12-16958-s006.docx]

Till now, most researches on CAFs biomarkers for breast cancer have been limited to a few pan-cellular markers and comprehensive analysis of the complex effects of CAFs on the prognostic and therapeutic response of breast cancer has not been reported. This study conducted a systematic analysis to assess the relationship between CAF biomarkers and the prognosis of breast cancers to provide more depth insights and offer stronger support for tailored individualized therapy.
