# Supplementary material for: The relationship between cancer associated fibroblasts biomarkers and prognosis of breast cancer: a systematic review and meta-analysis
Source: PeerJ. 2024 Feb 23;12:e16958. doi: 10.7717/peerj.16958 (PMC10896086; doi:10.7717/peerj.16958)
Supplement: Supplemental Information 13 [file peerj-12-16958-s013.docx]

**Supplementary Table S1** Summary of study characteristics of all included articles

| Research | Year | Type of tumor | Patients' no | Tumor stage | Cut-off value | fibroblast: (H/L) | Marker | Median follow-up (months) | Clinical outcome | Quality score (NOS) | Univariate | | Multivariate | |
| --- | --- | --- | --- | --- | --- | --- | --- | --- | --- | --- | --- | --- | --- | --- |
|  |  |  |  |  |  |  |  |  |  |  | HR | 95% CIs | HR | 95% CIs |
| Muchlińska, A. | 2022 | luminal | 85 | Stage Ⅰ-Ⅲ | index score >the upper quartile (Q3) of the whole group | 11/74 | α-SMA | NR | OS | 7 | 4.73 | 1.13-19.89 |  |  |
| Tanaka, Y. | 2021 | HR+ /HER2- | 304 | NR | ≥10% of the fibromatous tumor stroma | 27/142 | PDPN | 80.0 (5-143) months | DFS | 7 | 3.38 | 1.31-8.72 |  |  |
|  |  |  |  |  |  |  |  |  | DSS |  | 27.02 | 3.25-224.66 | 37.76 | 3.36-423.93 |
| Strell, C. | 2019 | DCIS | 351 | NR | IHC score >2 | 68/283 | PDGFR-α | NR | DFS | 7 | 0.85 | 0.49-1.49 | 0.95 | 0.51-1.77 |
|  |  |  | 360 | NR |  | 202/158 | PDGFR-β |  |  |  | 1.37 | 0.90-2.08 | 1.41 | 0.88-2.24 |
| Eiro, N. | 2019 | invasive | 246 | T1 or T2 | >10% positive cells | 175/71 | MMP11 | a minimum follow-up of ten years | RFS | 7 | 3.01 | 1.84-4.92 |  |  |
| Zhou, J. | 2018 | TNBC | 278 | NR | Immunostaining intensity × Response rate score 2-9 | 146/132 | FAP | 87 (8-130) months | DFS | 7 |  |  | 2.50 | 1.53-4.08 |
|  |  |  |  |  |  |  |  |  | DSS |  |  |  | 3.04 | 1.80-5.15 |
|  |  |  |  |  |  |  | α-SMA |  | DFS |  |  |  | 2.48 | 1.22-5.04 |
|  |  |  |  |  |  |  |  |  | DSS |  |  |  | 2.83 | 1.37-5.84 |
| Yang, Z. | 2017 | ductal | 150 | NR | IHC score 2-3 | 58/92 | FSP-1 | 68 (2-108) months | OS | 8 | 0.44 | 0.22-0.88 |  |  |
|  |  |  |  |  |  | 108/42 | α-SMA |  |  |  | 0.39 | 0.14-1.09 |  |  |
| Cai, D. | 2017 | invasive | 164 | Stage Ⅰ-Ⅲ | ≥10% of the fibromatous tumor stroma | 72/92 | PDPN | 42 (1-84) months | DFS | 7 |  |  | 3.93 | 1.52-10.17 |
| Amornsupak, K. | 2017 | IDC | 66 | NR | IHC score ≥3 | 39/27 | α-SMA | 65.23-87.7 months | MFS | 8 | 13.05 | 1.72-98.89 | 9.89 | 1.09-89.43 |
| Egeland, E.V. | 2016 | Early-stage | 291 | pT1N0 or pT2pN0G1 | IHC >1% stained tumor cells | 44/247 | FSP-1 | 88.8 (7.2-124.8) months | MFS | 8 | 0.37 | 0.17-0.79 |  |  |
|  |  | Early-stage |  |  |  |  |  |  | OS |  | 0.44 | 0.24-0.82 |  |  |
| Park, C. K. | 2016 | invasive | 524 | NR | IHC score ≥2 | 101/423 | PDPN | NR | OS | 8 |  |  | 2.10 | 0.89-4.98 |
|  |  |  |  |  |  |  |  |  | DFS |  |  |  | 3.46 | 1.41-8.48 |
|  |  |  |  |  |  | 340/184 | FSP-1 |  | DFS |  |  |  | 2.47 | 1.11-5.46 |
|  |  |  |  |  |  |  |  |  | OS |  |  |  | 0.58 | 0.27-1.22 |
|  |  |  | 642 |  |  | 153/489 | PDGFR-β |  | OS |  |  |  | 1.71 | 0.23-12.63 |
|  |  |  |  |  |  |  |  |  | DFS |  |  |  | 1.72 | 0.25-12.14 |
| Kim, H. M. | 2016 | Malignant phyllodes tumor | 16 | NR | IHC score >1 | 10/6 | PDGFR-β | NR | OS | 7 | 0.03 | 0.00-128.68 |  |  |
|  |  |  |  |  |  | 5/11 | PDGFR-α |  |  |  | 0.44 | 0.03-6.15 |  |  |
| Park, S. Y. | 2015 | BC | 642 | T1-T3 | IHC score ≥2 | 153/489 | PDGFR-β | NR | OS | 8 | 1.31 | 0.31-5.57 |  |  |
|  |  |  |  |  |  |  |  |  | DFS |  | 1.54 | 0.33-7.32 |  |  |
|  |  | Luminal B | 152 |  |  | 5/147 | FAP |  | OS |  | 0.01 | 0.00-3.52 |  |  |
|  |  | Invasive | 642 |  |  | 189/453 | FSP-1 |  | OS |  | 0.50 | 0.24-1.02 |  |  |
|  |  | Invasive |  |  |  |  |  |  | DFS |  | 0.98 | 0.46-2.11 |  |  |
|  |  | Luminal A | 275 |  |  | 40/235 | PDPN |  | OS |  | 0.16 | 0.02-1.57 |  |  |
| Martinez, L. M. | 2015 | Early-stage | 53 | NR | ≥10% of the stroma /HPF | 23/30 | FSP-1 | NR | OS | 6 | 0.32 | 0.01-10.42 |  |  |
| Jung, Y. Y. | 2015 | IDC | 642 |  | IHC score ≥2 | 23/619 | PDGFR-β | 68.3±30.1 months | OS | 7 | 1.68 | 0.27-10.65 |  |  |
|  |  |  |  |  |  |  |  |  | DFS |  | 1.74 | 0.27-11.18 |  |  |
|  |  |  |  | Stage Ⅰ-Ⅲ |  | 189/453 | FSP-1 |  | OS |  | 2.26 | 1.29-3.95 |  |  |
|  |  |  |  |  |  |  |  |  | DFS |  | 2.53 | 1.40-4.59 |  |  |
| Eiró, N. | 2015 | IDC | 107 | T1 or T2 | ≥10% of the stroma /HPF | 46/61 | TIMP-2 | 187/52 months | RFS | 7 | 5.10 | 3.00-8.80 |  |  |
|  |  |  |  |  |  |  |  |  | OS |  | 4.40 | 2.30-8.20 |  |  |
|  |  |  | 106 |  |  | 74/32 | MMP11 |  | RFS |  | 4.00 | 2.00-2.80 |  |  |
|  |  |  |  |  |  |  |  |  | OS |  | 4.00 | 1.90-100 |  |  |
|  |  |  | 107 |  |  | 57/50 | MMP13 |  | RFS |  | 1.70 | 1.04-3.00 |  |  |
|  |  |  |  |  |  |  |  |  | OS |  | 1.50 | 0.80-2.70 |  |  |
|  |  |  |  |  |  | 17/90 | MMP9 |  | RFS |  | 3.30 | 1.90-6.00 |  |  |
|  |  |  |  |  |  |  |  |  | OS |  | 3.90 | 2.00-7.80 |  |  |
| Pula, B. | 2013 | IDC | 104 | NR | immunoreaction score ＞3 | 74/30 | PDPN | 59.3±38.8 (1-125) months | OS | 6 | 2.08 | 0.80-5.43 |  |  |
| Pula, B. | 2013 | IDC | 257 | T1-T4, N0-N3 | Immunoreactive score 6-12 |  | PDPN | 63.21±38.54 (1-141) months | OS | 7 | 1.93 | 1.05-3.56 | 0.64 | 0.22-1.89 |
|  |  |  |  |  |  |  |  |  | EFS |  | 0.32 | 0.79-2.08 |  |  |
| Min, K. W. | 2013 | IDC | 192 | Stage Ⅰ-Ⅲ | Immunoreactive score >1 | 152/40 | MMP11 | NR | DFS | 7 | 1.67 | 0.86-3.22 |  |  |
|  |  |  |  |  |  |  |  |  | OS |  | 2.19 | 1.05-4.57 |  |  |
| Schoppmann, S. F. | 2012 | IDC | 367 | Stage Ⅰ-Ⅲ | ≥10% of the stroma /HPF | 33/334 | PDPN | NR | OS | 7 |  |  | 2.30 | 1.37-3.86 |
|  |  |  |  |  |  |  |  |  | DFS |  |  |  | 1.78 | 1.07-2.96 |
| Yamashita, M. etal | 2012 | IDC | 60 | Stage Ⅰ-Ⅲ | >8.48 % of the spindle-shaped cells/field area | 25/35 | α-SMA | 74.8 ± 19.3 months | OS | 6 | 0.63 | 0.02-17.14 |  |  |
|  |  |  | 60 |  |  |  |  |  | DFS |  | 0.30 | 0.04-2.23 |  |  |
| Busch, S. | 2012 | Invasive | 367 | N0-N3 | Immunoreactive score 2-3 | 283/84 | α-SMA | 69 months | RFS | 7 | 1.38 | 0.73-2.61 | 2.74 | 1.08-6.95 |
| Pula, B. | 2011 | IDC | 117 | T1-T4, N0-N3 | ≥10% of the stroma /HPF | 96/21 | PDPN | 58±38.55 (1-125) months | OS | 8 | 0.34 | 0.14-0.85 | 0.76 | 0.27-17.23 |
|  |  |  | 117 |  |  |  |  |  | DFS |  | 0.52 | 0.25-1.12 |  |  |
| Paulsson, J. | 2009 | BC | 289 | NR | IHC score 3 | 100/189 | PDGFR-β | 106 (0-207) months | RFS | 8 | 1.67 | 1.08-2.58 | 1.38 | 0.84-2.27 |
| Gonzalez, L. O. | 2009 | Luminal A | 48 | NR | IHC > median | 26/22 | TIMP-2 | 84/66 months | RFS | 7 | 4.80 | 1.90-11.90 |  |  |
|  |  | Basal like | 48 |  |  |  |  |  |  |  | 7.50 | 3.20-17.60 |  |  |
|  |  | Luminal A | 48 |  |  | 29/19 | MMP-11 |  |  |  | 4.90 | 1.60-14.90 |  |  |
|  |  | Basal like | 45 |  |  | 34/11 |  |  |  |  | 7.30 | 1.70-31.70 |  |  |
|  |  | Luminal A | 48 |  |  | 22/26 | MMP-13 |  |  |  | 2.70 | 1.10-6.40 |  |  |
|  |  | Basal like | 45 |  |  | 27/18 |  |  |  |  | 2.20 | 0.90-5.30 |  |  |
|  |  | Luminal A | 48 |  |  | 4/44 | MMP-9 |  |  |  | 3.10 | 1.00-9.20 |  |  |
|  |  | Basal like | 45 |  |  | 10/35 |  |  |  |  | 3.90 | 1.70-8.80 |  |  |
| Zhang, B. | 2008 | Invasive | 257 | N0-N3 | SI ≥6 | 125/132 | MMP-13 |  | OS | 7 | 1.00 | 0.79-1.26 |  |  |
| Surowiak, P. etal | 2007 | IDC | 45 | T1-T2, N0-N3, M0 | IHC score 2-3 | 28/17 | α-SMA | ≥96 months | DFS | 7 | 6.28 | 1.08-36.36 |  |  |
|  |  |  |  |  |  |  |  |  | OS |  | 1.23 | 0.17-0.79 |  |  |
| Ariga, N. | 2001 | IDC | 112 | NR | ≥10% of the stroma /HPF | 61/51 | FAP | NR | OS | 6 |  |  | 0.19 | 0.05-0.67 |
|  |  |  |  |  |  |  |  |  | DFS |  |  |  | 0.30 | 0.13-0.68 |

NOS = Newcastle-Ottawa Scale; breast cancer = BC; α-smooth muscle actin = α-SMA; podoplanin = PDPN; fibroblast activation protein = FAP; fibroblast-specific protein – 1 = FSP-1; platelet-derived growth factor receptor = PDGFR; tissue inhibitors of metalloproteinase-2 = TIMP-2; matrix metalloproteinase= MMP; overall survival = OS; disease-specific survival = DSS; progression-free survival = PFS; recurrence-free survival = RFS; disease-free survival = DFS; metastasis-free survival = MFS; hazard ratios = HRs; confidence intervals = CIs; immunohistochemistry = IHC; triple-negative breast cancers = TNBC; ductal carcinoma in situ = DCIS; tissue inhibitors of metalloproteinase = TIMP; human epidermal growth factor receptor 2 = HER2; The staining index = SI
